# Antioxidant Activities of Fractions of Polymeric Procyanidins from Stem Bark of *Acacia confusa*

**DOI:** 10.3390/ijms12021146

**Published:** 2011-02-15

**Authors:** Shu-Dong Wei, Hai-Chao Zhou, Yi-Ming Lin

**Affiliations:** Key Laboratory of the Ministry of Education for Coastal and Wetland Ecosystems, School of Life Sciences, Xiamen University, Xiamen 361005, China; E-Mails: weishudong2005@126.com (S.-D.W.); seapass2004@163.com (H.-C.Z.)

**Keywords:** *Acacia confusa*, stem bark, polymeric procyanidins, fractionation, antioxidant activity, MALDI-TOF MS, RP-HPLC

## Abstract

The polymeric procyanidins extracted from *Acacia confusa* stem bark were fractionated with a step gradient of water, methanol and acetone on a Sephadex LH-20 column. The antioxidant activity of the collected fractions was investigated by the 2,2-diphenyl-1-picrylhydrazyl (DPPH) free radical scavenging and ferric reducing/antioxidant power (FRAP) assays. All fractions possessed potent antioxidant activity with the highest activity observed for fraction F9. The matrix-assisted laser desorption/ionization time-of-flight mass spectrometry (MALDI-TOF MS) and reversed-phase high performance liquid chromatography (RP-HPLC) analyses suggested that the collected fractions consisted primarily of oligomeric and polymeric procyanidins, with different polymer ranges and most abundant polymer size. For each fraction, catechin and epicatechin were present as both terminal and extension units, and epicatechin was the major component in the extended chain. The mean degree of polymerization (mDP) of each fraction differed, ranging from 1.68 (fraction F2) to 17.31 (fraction F11). There was a relationship between antioxidant activity (IC50/DPPH and FRAP) and mDP (*R*^2^*_DPPH_* = 0.861, *P* = 0.006 and *R*^2^*_FRAP_* = 0.608, *P* = 0.038), respectively. However, the highest antioxidant activity of fraction (F9) was not coincident with the maximum mDP of fraction (F11).

## 1. Introduction

**Figure 1.**
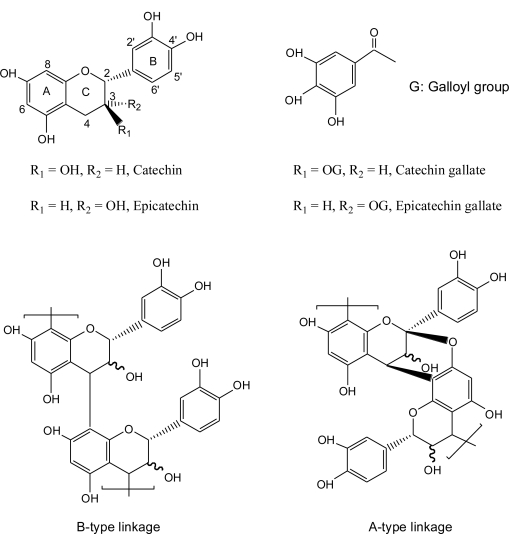
Chemical structures of monomeric flavan-3-ols and B-type linkage and A-type linkage procyanidins polymers.

Procyanidins, the major class of proanthocyanidins, yield cyanidin upon oxidation under strongly acidic conditions [1,2]. They consist mainly of (+)-catechin and/or (−)-epicatechin and their 3-*O*-gallates linked mainly through C4–C6 and/or C4–C8 bonds (B-type procyanidins) (Figure 1) [3,4]. The flavan-3-ol units can also be doubly linked by a C4–C8 bond and an additional ether bond formed between C2 and O7 (A-type procyanidins) [5,6].

Procyanidins are widely present in fruits, seeds, leaves, flowers and bark of many plants [7,8]. They possess a broad spectrum of biological, pharmacological and chemoprotective properties against free radicals and oxidative stress, such as antibacterial, antiviral, anti-inflammatory, anti-allergic and vasodilatory functions [9–12], as well as inhibiting lipid peroxidation, platelet aggregation and capillary permeability and fragility, and modulating the activity of enzyme systems, including cyclo-oxygenase and lipo-oxygenase [13]. Procyanidins have been considered as functional ingredients in botanical and nutritional supplements.

*Acacia confusa* is traditionally used as a medicinal plant [14]. An aqueous extract of *A. confusa* leaves was used for wound healing and anti-blood-stasis in Taiwan [15]. The stem bark of *A. confusa* is rich in procyanidins [16], and its crude extracts showed excellent antioxidant activities [17,18]. The size of procyanidins, expressed as degree of polymerization (DP), is one of the most important properties [5]; some previous studies showed that the DP increased the antioxidant capacity [19,20]. Due to the heterogeneous character of proanthocyanidins, the structural elucidation of these compounds (especially the higher polymers) is difficult and less is known regarding structure-activity relationships [21,22]. In order to describe the influence of the procyanidins DP on its antioxidant activity and filter out the best antioxidant performance fraction, it is necessary to establish an efficient, reliable separation method to separate the polymers according to their molecular weight.

Separations of procyanidins *i.e*., using normal phase [23], reversed phase [24] and size exclusion [25] models of liquid chromatography only allowed good separation of oligomeric procyanidins, and became ineffective for highly polymerized procyanidins [26]. Sephadex LH-20 has been widely used for proanthocyanidins chromatography since 1974 [27] and the separation methods based on it are more likely to be reproducible [28]. In the present study, the crude procyanidins extract from the stem bark of *A. confusa* was fractionated on a Sephadex LH-20 column using a gradient of water, methanol, and acetone. The obtained fractions were analyzed by MALDI-TOF MS and RPHPLC analyses. Meanwhile, the free radical scavenging capacities and ferric reducing/antioxidant powers of each fraction were also discussed.

## 2. Results and Discussion

### 2.1. Antioxidant Activities of Fractions F1-F11

The antioxidant activity of each fraction (F1–F11) was evaluated by measurement of their DPPH radical scavenging activities and ferric reducing antioxidant powers.

DPPH is a stable radical and has been accepted as a tool for estimating free radical scavenging activities of the *A. confusa* leaf, bark, twig, and branch extracts [29–31]. This method is based on the reduction of DPPH in methanol solution in the presence of a hydrogen-donating antioxidant due to the formation of the nonradical form DPPH-H. There was a significant linear relationship (*R*^2^ ≥ 0.996) between the concentrations of each fraction in the DPPH solution and the inhibition percentages. Using these linear equations, each fraction concentration providing 50% inhibition (IC_50_) was calculated. Lower IC_50_ value reflects better DPPH radical scavenging activity [32]. By comparing the IC_50_ value of fractions F1–F11 and the reference antioxidant compounds (BHA and ascorbic acid), F9 had the highest radical scavenging ability (81.91 ± 0.60 μg/mL) (Table 1). The radical scavenging ability of F9 was almost six-fold that of F1. The DPPH radical scavenging abilities of F5-F8 were also higher than that of ascorbic acid and BHA. The scavenging effect on the DPPH radical followed the order: F9 > F8 ≈ F7 ≈ F5 > F6 > F4 ≈ ascorbic acid > F10 ≈ F3 ≈ BHA > F11 > F2 > F1.

The ferric reducing antioxidant assay is based on the reduction of TPTZ-Fe (III) to the TPTZ-Fe (II) complex by a reductant at low pH [33]. The procedure is relatively simple and easy to standardize. It has been used frequently in the assessment of antioxidant activity of various fruits, vegetables and some biological samples [34–36]. The antioxidant ability of each fraction was estimated by FRAP values, which is expressed in ascorbic acid equivalents. The FRAP values for fraction F1–F11 ranged from 1.33 ± 0.06 to 6.33 ± 0.04 mmol AAE/g, with the highest in F9 and the lowest in F1, respectively (Table 1). In brief, the reducing power of the fraction and standard were found in the following order: F9 > F5 ≈ F4 > F8 ≈ F7 ≈ F3 > F6 ≈ BHA > F10 ≈ F2 > F11 > F1.

According to the results mentioned above, the antioxidants were more abundantly distributed in fractions F4–F9 than in other fractions. In addition, compared with the antioxidant activities of the total *A. confusa* stem bark procyanidins before separation (IC_50_ = 87.85 ±0.52 μg/mL, FRAP = 5.89 ±0.14 mmol AAE/g) [16], prior separation of the procyanidins can filter out the best antioxidant performance fraction (F9) as antioxidants. Furthermore, to identify and characterize the antioxidants in *A. confusa* stem bark and demonstrate the relationship between the structure of polymeric tannins and their antioxidant activity, the active fractions were analyzed by MALDI-TOF MS and acid-catalyzed degradation analyses.

### 2.2. MALDI-TOF MS Analysis

The MALDI-TOF MS is considered a sensitive and powerful tool for the analysis of both synthetic and natural polymers [37–40]. Because of its soft ionization energy, high ion transmission yield, highly contaminant tolerance, and because it produces only a singly charged molecular ion for each parent molecular attributes [41,42], the simultaneous determination of masses in complex mixtures of low and high molecular weight compounds can be achieved [43].

**Figure 2.**
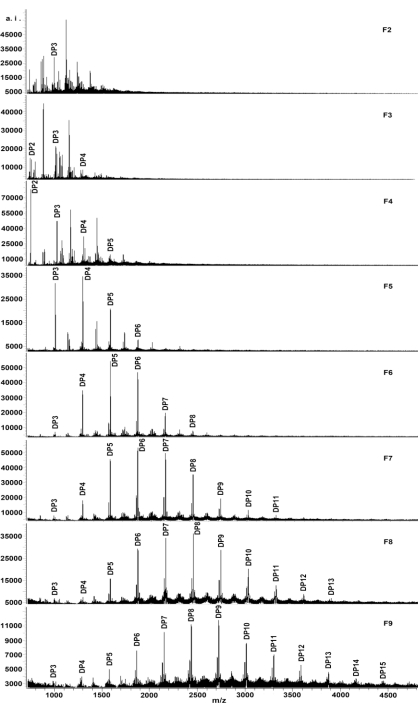
MALDI-TOF positive ion reflectron mode mass spectra of fractions F2–F9 by fractionation of the crude procyanidins extract from *A. confusa* stem bark.

Figure 2 shows the MALDI-TOF MS spectra of fractions F2–F9, recorded as Cs^+^ adducts in the positive ion reflectron mode. Due to the compounds in fraction F1 eluting with water being mainly sugars and the signal-to-noise ratio being too weak in the spectrum, this fraction is not included in the subsequent analysis. Fractions F10 and F11 could not be ionized because of the high mDP of the polymers. Some authors [44,45] suggested that the detection of high molecular weight tannins is less sensitive compared with low molecular weight species. Lower masses (from the matrix) might saturate the detector, preventing the detection of polymeric tannins with higher masses [46].

The MALDI-TOF mass spectra of fractions F2–F9 are simple in appearance, with peaks grouped at intervals corresponding to difference in DP. The results obtained in the present study indicated a series of peaks with distances of 288 Da, corresponding to a mass difference of one catechin/epicatechin between each polymer. Prolongation of polymeric tannins is due to addition of catechin/epicatechin monomers. The polymeric tannins of *A. confusa* stem bark consisted primarily of procyanidin. This is in accordance with the observation of Wei *et al*. 2010 [16].

Procyanidin oligomers from many plants have been identified by MALDI-TOF MS [47,48]. An equation was formulated to calculate the expected polymeric procyanidins molecular weight. The equation is *M* = 290 + 288*a* + 133, where *M* represents the calculated mass, 290 is the molecular weight of the terminal catechin/epicatechin unit, *a* is the degree of polymerization contributed by the catechin/epicatechin extending unit, and 133 is the weight of cesium [49]. A series of ions corresponding to cesium adducts of procyanidin oligomers and polymers from the DP 2 through to the DP 15 were observed in fractions F2–F9 (Table 2).

Table 3 summarized the range of polymers and the most abundant polymer size in the eight collected fractions F2–F9. The overall polymer range of the mass spectra was gradually increased from the fractions F2–F9. Large molecular size of polymeric procyanidins was detected in the fraction F9, up to DP 15, which was not apparent in the spectrum of *A. confusa* stem bark reported by Wei *et al*. 2010 [16]. It is well demonstrated that prior size separation of *A. confusa* stem bark procyanidins allows more to be learned about the molecular weight distribution of polymeric procyanidins and improves the detection of large polymers.

### 2.3. Thiolysis of Fractions F2–F11

**Figure 3.**
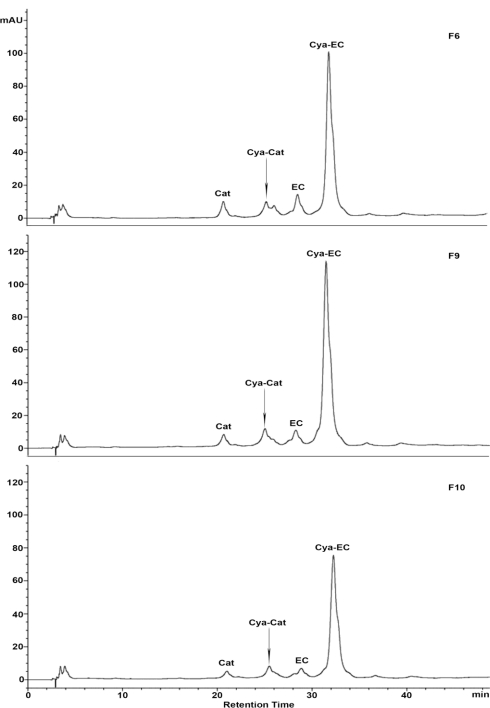
Reversed phase HPLC chromatograms of fraction F6, F9, and F10 degraded in the presence of cysteamine; Cat, (+)-catechin; EC, (−)-epicatechin; Cya-Cat, 4 β-(2-aminoethylthio) catechin; Cya-EC, 4β-(2-aminoethylthio) epicatechin.

Depolymerization reactions in the presence of nucleophiles have been proven to be an efficient method for the structure analysis of procyanidins [50]. To determine each fraction subunit composition and mDP, the collected samples (F2–F11) were analyzed by acid catalysis in the presence of cysteamine as nucleophile, which was preferred to toluene-*a*-thiol due to being more user-friendly and much less toxic [51]. In thiolysis reactions, all the extension subunits of procyanidins are attacked by cysteamine to form the 4-(2-aminoethylthio) flavan-3-ols. Only the terminal unit is released as the free flavan-3-ol [5]. The extension units and terminal units of procyanidins can be distinguished by reversed phase HPLC analysis (Figure 3).

Figure 3 shows the chromatograms of polymeric procyanidins in fractions F6, F9, and F10. The major product observed was the 4β-(2-aminoethylthio) epicatechin (Cya-EC) along with a small amount of (+)-catechin (Cat), (−)-epicatechin (EC), and 4β-(2-aminoethylthio) catechin (Cya-Cat). The result suggested that catechin and epicatechin occur as both terminal and extension units, and that epicatechin was the major component in the extended chain of the polymeric procyanidins from *A. confusa* stem bark.

The mDP of the proacyanidin fractions was calculated by comparing the peak areas based on the following equation (Table 3):

$$\text{mDP}=\frac{\text{total area of cystemine derivative of catechin and cysteamine derivative of epicatechin}}{\text{total area of catechin and epicatechin}}+1$$

Interestingly, the elution order was coincident with the mDP of procyanidins. The mDP of each fraction was different, ranging from 1.68 to 17.31, with the maximum in the last fraction F11 and the minimum in the fraction F2.

Most of the activities of procyanidins, including the antioxidant activity, are generally recognized to be largely dependent on their structure, particularly their degree of polymerization [52,53]. In the present study, the regression model of the relationship between antioxidant activity (IC_50/DPPH_ and FRAP) and mDP were as follows: IC_50/DPPH_ = −0.028 mDP^3^ + 1.452 mDP^2^ − 18.146 mDP + 151.518 (*R*^2^ = 0.861, *P* = 0.006) and FRAP = −0.022mDP^2^ + 0.328 mDP + 4.629 (*R*^2^ = 0.608, *P* = 0.038), respectively. The highest antioxidant activity of fraction (F9) was not coincident with the max mDP of fraction (F11) (Tables 1 and 3). The tendency was similar to the reports by Jerez *et al*. [54], who found an increase up to 6.5 mDP and then a fall (9.2–14.6 mDP) of the antioxidant activity of procyanidins from *Pinus radiata* bark.

## 3. Experimental Section

### 3.1. Chemicals and Plant Materials

The solvents acetone, petroleum ether, ethyl acetate and methanol were of analytical reagent (AR) purity grade. The trifluoroacetic acid (TFA) and acetonitrile were of HPLC grade. 2,2-diphenyl-1-picrylhydrazyl (DPPH), 2,4,6-tripyridyl-S-triazine (TPTZ), cysteamine hydrochloride, ascorbic acid, butylated hydroxyanisole (BHA), and cesium chloride were purchased from Aldrich (USA). (−)-Epicatechin (EC) and (+)-catechin (Cat) were purchased from Sigma (USA). Sephadex LH-20 was purchased from Amersham (USA). The stem bark of *A. confusa* were collected from Xiamen Botanical Garden, Fujian province, China in December 2009 and immediately freeze dried and ground.

### 3.2. Extraction and Fractionation of Procyanidin Polymers

Freeze-dried stem bark powder (35 g) was extracted thrice with 7:3 (v/v) acetone-water solution (3 × 250 mL) at room temperature. The extracts were centrifuged at 3000 g for 15 min and collected, and the acetone was removed under reduced pressure by use of a rotary evaporator at 38 °C. The remaining aqueous fraction was defatted with petroleum ether (3 × 150 mL), and then extracted with ethyl acetate (3 × 150 mL). Two fractions were obtained: an organic fraction and an aqueous fraction which contained mainly polymers. The solvent (water saturated with ethyl acetate) was eliminated from the aqueous fraction and lyophilized to obtain crude tannins extract.

The crude tannins extract (500 mg/5 mL of 50% methanol) was applied to a Sephadex LH-20 column (50 × 1 cm i.d.). The column was eluted with water (150 mL, F1), methanol-water (40:60, v/v; 100 mL, F2), methanol-water (60:40, v/v; 50 mL, F3 and 50 mL, F4), methanol-water (75:25, v/v; 100 mL, F5), methanol-water (90:10, v/v; 50 mL, F6), acetone-methanol-water (10:80:10, v/v/v; 50 mL, F7 and 50 mL, F8), acetone-methanol-water (20:65:15, v/v/v; 50 mL, F9), acetone-methanol-water (30:40:30, v/v/v; 50 mL, F10), and finally acetone-water (70:30, v/v; 200 mL, F11). The fractions were concentrated *in vacuo* (38 °C) and freeze-dried to give eleven powders with approximate weights of 183 mg, 11.7 mg, 16.1 mg, 22.3 mg, 47.2 mg, 23 mg, 50.3 mg, 39.5 mg, 33.7 mg, 22.3 mg, and 15.6 mg for F1–F11, respectively.

### 3.3. DPPH Radical Scavenging Activity

The DPPH free radical scavenging activity of each fraction was determined according to the method of Braca *et al*. 2001 [55]. 0.1 mL of various concentrations of each freeze-dried sample at different concentrations (15.63–125 μg/mL) was added to 3 mL of DPPH solution (0.1 mM in methanolic solution). An equal amount of methanol and DPPH served as control. After the mixture was shaken and allowed to stand at ambient temperature for 30 min, the absorbance at 517 nm was measured. Lower absorbance of the reaction mixture indicates higher free radical scavenging activity. The IC_50_ value, defined as the amount of antioxidant necessary to decrease the initial DPPH concentration by 50%, was calculated from the results and used for comparison. The capability to scavenge the DPPH radical was calculated by using the following equation:

$$\text{DPPH scavenging effect}(\%)=[({\text{A}}_{1}-{\text{A}}_{2})/{\text{A}}_{1}]\times 100$$

where A_1_ = the absorbance of the control reaction; A_2_ = the absorbance in the presence of the sample. BHA and ascorbic acid were used as standards.

### 3.4. Ferric Reducing/Antioxidant Power (FRAP) Assay

FRAP assay is a simple and reliable colorimetric method commonly used for measuring the total antioxidant capacity [56]. In brief, 3 mL of prepared freshly FRAP reagent was mixed with 0.1 mL of test sample or methanol (for the reagent blank). The FRAP reagent was prepared from 300 mmol/L acetate buffer (pH 3.6), 20 mmol/L ferric chloride and 10 mmol/L TPTZ made up in 40 mmol/L hydrochloric acid. All the above three solutions were mixed together in the ratio of 25:2.5:2.5 (v/v/v). The absorbance of reaction mixture at 593 nm was measured spectrophotometrically after incubation at 25 °C for 10 min. The FRAP values, expressed in mmol ascorbic acid equivalents (AAE)/g dried tannins, were derived from a standard curve.

### 3.5. MALDI-TOF MS Analysis

The MALDI-TOF MS spectra were recorded on a Bruker Reflex III instrument (Germany). The irradiation source was a pulsed nitrogen laser with a wavelength of 337 nm, and the duration of the laser pulse was 3 ns. In the positive reflectron mode, an accelerating voltage of 20.0 kV and a reflectron voltage of 23.0 kV were used. 2,5-Dihydroxybenzoic acid (DHB, 10 mg/mL 30% acetone solution) was used as the matrix. The sample solutions (10 mg/mL 30% acetone solution) were mixed with the matrix solution at a volumetric ratio of 1:3. The mixture (1 μL) was spotted to the steel target. Amberlite IRP-64 cation-exchange resin (Sigma-Aldrich, USA), equilibrated in deionized water, was used to deionize the analyte-matrix solution thrice. Cesium chloride (1.52 mg/mL) was mixed with the analyte-matrix solution (1:3, v/v) to promote the formation of a single type of ion adduct ([M+Cs]^+^) [57].

### 3.6. Thiolysis Reaction and HPLC Analysis

Thiolysis was carried out according to the method of Torres and Lozano 2001 [58]. The chosen fraction solution (4 mg/mL in methanol) was prepared. A sub-sample (50 μL) was placed in a vial and to this hydrochloric acid in methanol (3.3%, v/v; 50 μL) and cysteamine hydrochloride in methanol (50 mg/mL, 100 μL) was added. The solution was heated at 40 °C for 30 min, and cooled to room temperature. Thiolysis reaction media (20 μL) filtrated through a membrane filter with an aperture size of 0.45 μm was analyzed by RP-HPLC.

The high performance liquid chromatographer was an Agilent 1200 system (USA). The thiolysis media were further analyzed using LC/MS (QTRAP 3200, USA) with a Hypersil ODS column (4.6 mm × 250 mm, 2.5 μm) (China). The mobile phase was composed of solvent A (0.5% v/v trifluoroacetic acid (TFA) in water) and solvent B (0.5% v/v TFA in acetonitrile). The gradient condition was: 0–5 min, 3% B (isocratic); 5–15 min, 3%–9% B (linear gradient); 15–45 min, 9%–16% B (linear gradient), 45–60 min, 16%–60% B (linear gradient). The column temperature was ambient and the flow-rate was set at 1 mL/min. Detection was at a wavelength of 280 nm and the UV spectra were acquired between 200–600 nm. Degradation products were identified on chromatograms according to their relative retention times and LC/MS.

### 3.7. Statistical Analysis

All data were expressed as means ± standard deviation of three independent determinations. One-way analysis of variance (ANOVA) was used, and the differences were considered to be significant at *P* < 0.05. All statistical analyses were performed with SPSS 13.0 for windows.

## 4. Conclusions

The use of water, methanol and acetone step gradient on Sephadex LH-20 column is a good method to fractionate the polymeric procyanidins of *A. confusa* stem bark according to the degree of polymerization. The collected fractions all possessed potent antioxidant activity with the best result achieved for F9. The MALDI-TOF MS and RP-HPLC analyses suggested that the fractions consisted primarily of oligomeric and polymeric procyanidins, with different polymer ranges and most abundant polymer size. For each fraction, catechin and epicatechin were present as both terminal and extension units, and epicatechin was the major component in the extended chain. The method described can easily be applied to filter out the fraction with the strongest antioxidant activity, to demonstrate well the effectiveness of prior size separation in improving mass spectra detection of the procyanidins, and also to facilitate elucidation of structure-activity relationships in assays of procyanidins.

## Figures and Tables

**Table 1.** Antioxidant activities of each fraction eluted from the Sephadex LH-20 column using the DPPH free radical scavenging assay and the ferric reducing antioxidant power (FRAP) assay.

**Fractions**	**Antioxidant Activity**	
	**IC_50/DPPH_ (μg/mL)***^a^*	**FRAP (mmol AAE/g)***^b^*
F1	450.24 ± 5.21 ^a^	1.33 ± 0.06 ^g^
F2	136.38 ± 4.01 ^b^	4.20 ± 0.09 ^e^
F3	116.64 ± 1.83 ^d^	5.55 ± 0.12 ^c^
F4	99.12 ± 0.77 ^e^	6.09 ± 0.06 ^b^
F5	90.74 ± 0.86 ^g^	6.13 ± 0.02 ^b^
F6	93.88 ± 0.44 ^f^	5.34 ± 0.06 ^d^
F7	89.84 ± 0.74 ^g^	5.52 ± 0.07 ^c^
F8	87.42 ± 1.82 ^g^	5.50 ± 0.06 ^c^
F9	81.91 ± 0.60 ^h^	6.33 ± 0.04 ^a^
F10	115.23 ± 0.94 ^d^	4.29 ± 0.05 ^e^
F11	129.72 ± 1.77 ^c^	3.97 ± 0.04 ^f^
Ascorbic acid	101.96 ± 1.84 ^e^	-
BHA	116.91 ± 0.97 ^d^	5.18 ± 0.11 ^d^

a The antioxidant activity was evaluated as the concentration of the test sample required to decrease the absorbance at 517 nm by 50% in comparison to the control;

b FRAP values was expressed in mmol ascorbic acid equivalent/g sample in dry weight; BHA: Butylated hydroxyanisole. Values are expressed as mean of duplicate determinations ± standard deviation; Different letters in the same column show significant differences from each other at *P* < 0.05 level.

**Table 2.** Summary of observed [M+Cs]^+^ ions of polymeric procyanidin fractions from *A. confusa* stem bark for MALDI-TOF mass spectra.

**Polymer**	**Observed [M+Cs]^+^**							
	**F2**	**F3**	**F4**	**F5**	**F6**	**F7**	**F8**	**F9**
DP2	--	711.21	711.13	--	--	--	--	--
DP3	999.25	999.29	999.20	999.24	999.26	999.43	999.37	999.36
DP4	--	1287.41	1287.26	1287.30	1287.33	1287.40	1287.33	1287.38
DP5	--	--	1575.35	1575.36	1575.38	1575.53	1575.51	1575.62
DP6	--	--	--	1864.46	1864.45	1864.63	1863.59	1863.57
DP7	--	--	--	--	2152.54	2152.70	2152.60	2152.60
DP8	--	--	--	--	2440.63	2440.78	2440.70	2440.63
DP9	--	--	--	--	--	2728.87	2728.76	2728.71
DP10	--	--	--	--	--	3016.93	3017.69	3017.10
DP11	--	--	--	--	--	3304.86	3305.39	3305.81
DP12	--	--	--	--	--	--	3593.99	3593.81
DP13	--	--	--	--	--	--	3881.56	3881.59
DP14	--	--	--	--	--	--	--	4169.72
DP15	--	--	--	--	--	--	--	4457.63

**Table 3.** Comparison of the mean degree of polymerization (mDP) estimated by acid catalysis and MALDI-TOF mass spectrometry.

**Fractions**	**mDP by Thiolysis**	**Polymer Range in MALDI**	**Most Intense Polymer in MALDI**
F2	1.68 ± 0.05 ^j^	3	3
F3	2.13 ± 0.16 ^i^	2–4	3
F4	2.71 ± 0.06 ^h^	2–5	2
F5	5.46 ± 0.09 ^g^	3–6	4
F6	7.37 ± 0.08 ^f^	3–8	5
F7	9.49 ± 0.11 ^e^	3–11	6
F8	9.80 ± 0.09 ^d^	3–13	8
F9	10.65 ± 0.13 ^c^	3–15	9
F10	15.44 ± 0.06 ^b^	-	-
F11	17.31 ± 0.16 ^a^	-	-

Different letters in the same column show significant differences from each other at *P* < 0.05 level.
